# Central Nervous System Infection with *Histoplasma capsulatum*

**DOI:** 10.3390/jof5030070

**Published:** 2019-07-24

**Authors:** James Riddell, L. Joseph Wheat

**Affiliations:** 1 Division of Infectious Diseases, University of Michigan Health System, 1500 E. Medical Center Drive, University Hospital South F4131, Ann Arbor, MI 48109, USA; 2 MiraVista Diagnostics, Indianapolis, IN 46241, USA

**Keywords:** Histoplasmosis, meningitis, central nervous system infection

## Abstract

Histoplasmosis is an endemic fungal infection that may affect both immune compromised and non-immune compromised individuals. It is now recognized that the geographic range of this organism is larger than previously understood, placing more people at risk. Infection with *Histoplasma capsulatum* may occur after inhalation of conidia that are aerosolized from the filamentous form of the organism in the environment. Clinical syndromes typically associated with histoplasmosis include acute or chronic pneumonia, chronic cavitary pulmonary infection, or mediastinal fibrosis or lymphadenitis. Disseminated infection can also occur, in which multiple organ systems are affected. In up to 10% of cases, infection of the central nervous system (CNS) with histoplasmosis may occur with or without disseminated infection. In this review, we discuss challenges related to the diagnosis of CNS histoplasmosis and appropriate treatment strategies that can lead to successful outcomes.

## 1. Introduction

Histoplasmosis was initially described in 1906 by Samuel T. Darling who was working as a pathologist in Panama. At that time, he described a case of a man from Martinique who had died of disseminated infection of the lung, liver, spleen, and lymph nodes [1]. On autopsy, Dr. Darling found dividing organisms of 1–4 microns in size in multiple organ systems, which were different from other infectious organisms known at the time. He proposed the name *Histoplasma capsulata* for this new entity, which he initially described as a parasite. It was subsequently determined that this organism is a dimorphic fungus now known as *Histoplasma capsulatum*, which grows as a yeast at warm temperatures and assumes a filamentous form with fruiting bodies at cooler temperatures. Histoplasmosis is known as an “endemic fungus” because it has been identified in certain geographical areas, such as the mid portion of the United States, Canada, Mexico, Central and South America, as well as, less commonly, Africa, Asia, and Europe [2]. When humans inadvertently inhale conidia produced by the fruiting bodies of *Histoplasma capsulatum*, infection may occur if these organisms are not first eliminated by the reticuloendothelial system. Defects in cell mediated immunity (either innate or acquired) place individuals at higher risk of severe infection, however, immune competent individuals are also at risk. The lung is the initial site of infection after inhalation. However, this organism can subsequently disseminate to multiple organ systems, including the central nervous system.

Infection of the central nervous system with histoplasmosis was first recognized in an infant in 1934 [3]. Autopsy studies performed in the 1950s at Vanderbilt University subsequently confirmed that infection of central nervous system structures could occur with fatal disseminated infection. Of 11 cases of disseminated histoplasmosis identified in this study, 6 were found to have infection of the central nervous system, with only 2 patients reported to have pre-morbid symptoms of meningitis [4]. Since that time, there have been numerous case reports and case series describing central nervous system infection with histoplasmosis. Overall, it is now estimated that 5–10% of cases of progressive disseminated histoplasmosis will have central nervous system (CNS) infection [5]. However, for the clinician, making a diagnosis of fungal meningitis can be challenging because symptoms may be subtle and fungal culture of cerebral spinal fluid (CSF) has poor sensitivity [6]. Furthermore, clinicians may not recognize that CNS infection is important to consider or be aware of specialized diagnostic testing that is available. If present, it is important to establish the diagnosis of CNS infection with histoplasmosis because it has a significant impact on treatment strategies. Newer diagnostic modalities, including antigen and improved antibody testing methods, now exist to aid clinicians in establishing if central nervous system infection with *Histoplasma capsulatum* is present. The prognosis for patients with this potentially fatal infection, which may relapse if incompletely treated, can be significantly improved with appropriate therapy and monitoring.

In this review, we discuss risk factors and the typical clinical presentation of CNS infection with histoplasmosis and focus on the effectiveness of various diagnostic modalities, including culture, serologic testing, and antigen testing. Antifungal management will then be reviewed, including discussion of the newer antifungals and the importance of laboratory monitoring to rule out relapse as patients are followed over time. The goal is to increase awareness of this rare but significant complication associated with histoplasmosis and to improve recognition of the various diagnostic tests and subsequent treatment strategies that currently exist to improve outcomes.

## 2. Clinical Presentation

### 2.1. Risk Factors

Individuals who reside in or have been within the expanding endemic area for *Histoplasma* [2] are at risk for infection, regardless of immune function or comorbidities. However, people with a suppressed immune system are at particularly high risk. HIV infection with associated AIDS, organ transplantation, or treatment with immune suppressive medications, such as corticosteroids or TNF-inhibitors, all have been shown to place individuals at higher risk of complications related to histoplasmosis [7,8,9,10]. Those who are at the extremes of age are also at risk (infancy or ages >54 years old). With regards to CNS infection, in the largest case series to date, 44% of patients had a diagnosis of AIDS, 13% had undergone organ transplantation, 14% were receiving immune suppressing medications, and 13% were over the age of 54 [11]. Notably, 16% had no underlying comorbidity or advanced age in this study. In an earlier study of 18 patients, 8 patients had no underlying immune suppression while 6 had AIDS and 4 had other causes for immune suppression [5].

### 2.2. Symptoms

One of the first major case series that described the clinical course of patients with CNS histoplasmosis was published in 1990 [5]. As part of recurrent large outbreaks of histoplasmosis in Indianapolis, IN, that had occurred over the preceding years, 18 cases of histoplasmosis with CNS involvement were identified, with 23 total episodes of infection when relapses were included. All patients in this study presented with at least one neurological symptom, which included headache (*n* = 8), altered mental status (*n* = 10) with coma (*n* = 4), stroke or focal neurological deficit (*n* = 8), and seizure (*n* = 5). Upon review of the available literature at that time, an altered level of cognitive function was present in 50% of 104 total cases reported. Other common neurological signs and symptoms of these collected patients of a literature review included headache (24%), cranial nerve deficit (19%), other focal neurological deficit (19%), or seizure (13%). The duration of symptoms in this study varied greatly. Six patients had symptoms for less than one month, 10 had symptoms from 2–6 months, and 12 for greater than 6 months. General groups of clinical presentation were identified in this study. Disseminated histoplasmosis with chronic meningitis was most common in 34 out of 77 or 39% of cases. Isolated *Histoplasma* meningitis, without involvement of other organ systems, was found in 25% of cases. It is likely that isolated infection of the CNS occurs as a result of disseminated infection without an adequate immune response within the protected space across the blood brain barrier. Space-occupying parenchymal brain lesions were found in 24% of cases [11].

In the largest case series to date, 77 patients with possible, probable, or proven CNS histoplasmosis were identified in multiple medical centers across the country [11]. In this study, the most common symptoms included headache in 60%, with altered mental status reported in 42%. Focal neurological deficits were identified in 30%. A smaller case series from Brazil demonstrated similar findings. Of 11 patients identified over a period from 1978 to 2003, 9 patients presented with headache, with 5 patients described as having mental status changes [12]. In general, symptoms tend to be subacute in nature. Of 72 patients with data available, 29% had symptoms for a range of 5 to 26 weeks, while 24% had a duration of less than 2 to 4 weeks [11]. Meningitis was a manifestation of relapsed disseminated histoplasmosis in 16% of patients (Table 1). Relapse of infection after initial response to therapy occurred most commonly in the setting of AIDS with advanced immune compromise and poor medical adherence.

### 2.3. Physical Exam Findings

Abnormalities upon physical exam tend to reflect findings observed in chronic meningitis or neurologic deficits associated with space-occupying mass lesions. In the first major case series and literature review, which analyzed 104 patients in total, 29% were described as having depressed consciousness and 22% had confusion. Cranial nerve deficits occurred in 19%, while other focal neurological deficits occurred in 16% of all patients. Meningismus was only found in 8% of patients [5]. Findings associated with disseminated histoplasmosis were also fairly common, as follows: Hepatomegaly in 30%, splenomegaly in 20%, lymphadenopathy in 11%, mucosal lesions in 15% and cutaneous lesions in 5%. In the more recent case series of 77 patients, 42% were found to have altered mental status upon exam, 30% had focal neurological deficits, 13% had neck rigidity, and 9% presented with visual impairment [11]. Other less common presentations, such as with hemichorea, have also been observed [13]. In many cases of *Histoplasma* meningitis, an alternative diagnosis is often considered based on physical findings and presentation. In one such case, Creutzfeldt–Jakob disease was initially considered, however, on autopsy, *Histoplasma* yeast forms were found in parenchymal brain tissue [14].

### 2.4. Diagnosis

The diagnosis of *Histoplasma* meningitis is often delayed by more than one month in up to 60% of cases, and some only at autopsy [5,11]. The main reason for delay is failure to suspect a fungal etiology. Many cases of central nervous system histoplasmosis are misdiagnosed and treated for other diseases, such as neurosarcoidosis, tuberculosis, toxoplasmosis, inflammatory diseases, and malignancy [11]. Some physicians believe that CNS histoplasmosis should only be considered in immunocompromised patients. In case series, 29% to 50% of patients were not immunocompromised and 16% to 36% had no recognized underlying condition predisposing them to dissemination [11,15,16].

*Histoplasma* meningitis often presents as a chronic lymphocytic meningitis. Large case series have shown that the duration of symptoms is less than 1 month in 30% [17] to 50% of cases, including 28% that presented with symptoms for 2 weeks or less [5,11]. Another cause for failure to suspect CNS histoplasmosis is that up to half of patients do not present with the expected CSF profile with lymphocytic pleocytosis [11]. In one series, CSF cell count, glucose, and protein were found to be normal in 17% of patients who were eventually diagnosed with CNS histoplasmosis. Leukocytosis greater than 5 cells/µL occurred in only 66% of patients. Lymphocytes were predominant in 50% and neutrophils in 11% of patients. Protein was greater than 50 mg/mL in 77% of patients and glucose was less than 40 mg/mL in 53% [11].

CNS imaging may also provide evidence for meningeal or parenchymal inflammation, but does not distinguish between other infectious and noninfectious etiologies. Computed tomography (CT) was performed in 28% of patients and magnetic resonance imaging (MRI) in 72% [11]. Imaging was normal in 28% of patients in this large case series [11]. The most common finding was mass lesions in 20% of patients. The next most common was ventricular enlargement (9%), followed by meningeal enhancement (8%). A T2 flare signal was seen in 7%, infarcts in 3%, other findings in 8%, and multiple abnormalities in 17% of patients.

In the most recent large case series, the diagnosis of CNS histoplasmosis was established less than 1 week following presentation in 11% of patients, 1–2 weeks in 17%, 2–4 weeks in 14%, 5–26 weeks in 31%, and more than 26 weeks in 27% of patients [11]. In most cases, the diagnosis can be determined based on detection of the *Histoplasma* antigen or anti-*Histoplasma* antibodies in the CSF [15]. In a recent study, the sensitivity for antigen detection in the CSF was 78% and the specificity was 97% (Table 2) [15]. Methods for antibody detection include immunodiffusion (ID), complement fixation (CF), and enzyme immunoassay (EIA) [15]. EIA for IgG and IgM anti-*Histoplasma* antibodies was more sensitive (82%) than ID (44%), complement fixation (CF) (50%), or a combination of ID and CF (51%). Specificity was 93% for the EIA and 96% for immunodiffusion and complement fixation combined [15]. In a retrospective study in which antigen testing and antibody EIA testing were performed in patients with CNS histoplasmosis, differences in sensitivity and specificity for these tests were observed in immunocompromised versus non-immunocompromised patients. A higher proportion of immunocompromised patients had positive antigen testing; however, fewer immunocompromised patients had positive ID or CF antibodies or EIA IgG or IGM antibodies detected [15].

*Histoplasma* antigen testing is available in the United States (US) at MiraVista Diagnostics using a laboratory developed test (MVista *Histoplasma* antigen quantitative EIA) [18] and other commercial laboratories using a kit developed by Immuno-Mycologics Inc. (IMMY), The *Histoplasma* galactomannan (HGM) EIA [19]. Antigen testing is not widely available outside of the US, including in Latin America, where histoplasmosis is common. IMMY’s *Histoplasma* HGM EIA is available in some parts of Latin America. A multicenter study in Brazil evaluated the IMMY Histoplasma HGM EIA in culture or pathology proven cases of histoplasmosis in patients with HIV/AIDS and reported its sensitivity to be 56% [20].

Other diagnostic tests, such as β-d-glucan detection using the Fungitell^®^ immunochromatographic assay, have also been studied in the CSF. This test was positive in CSF at ≥80 pg/mL in 50% of cases of CNS histoplasmosis and 20% of controls, which included other fungal infections, non-fungal meningitis, and noninfectious disorders [21]. Given the poor sensitivity and specificity, the β-d-glucan test is not considered to be useful for diagnosis of *Histoplasma* meningitis. Real time PCR assays are also being developed to detect *Histoplasma*. In one study, authors demonstrated 100% specificity and 73% sensitivity from 797 clinical specimens [22], however, more study needs to be completed before recommending PCR as a standard part of the diagnostic armamentarium. 

Antigen testing of urine and serum and antibody testing of serum are also important components of evaluating for the presence of CNS infection with histoplasmosis. Antigen was detected in the urine of 73% and serum of 50% of patients on whom testing was performed [11]. Anti-*Histoplasma* antibodies were detected in the serum of 59% of patients that were tested in this study. Antigen was detected in the urine or serum in 77% of cases in a different study [15]. Correlation of CSF antigen testing and urine antigen testing in the setting of CNS infection tends to be poor. For example in one study it was noted that, CSF was positive and urine was negative in 17% of 46 cases [15].

In addition to antigen and serological testing, fungal culture of CSF should be performed. As the sensitivity of CSF culture is poor, multiple samples may be needed to establish a diagnosis [5]. Culture of CSF was positive in only 19% of cases in the most recent large case series (Table 2) [11]. When investigating for the presence of CNS histoplasmosis, at least 10 mL of CSF should be cultured for fungus and the culture should be held for at least 6 weeks. More than one culture may be necessary to isolate the organism. Cytology of CSF is positive in less than 20% of cases (cytology positive in 2 of 11 patients in unreported portion of the data set) [11]. However, yeast form organisms of 1–4 microns in diameter is often observed on the histopathology of biopsied brain lesions (Table 2) [11]. In severely ill patients, the biopsy of brain lesions identified on imaging may be preferred over repeat CSF evaluation to obtain a definitive diagnosis more quickly (or when CSF cannot be safely obtained). When fungal infection is suspected, a silver stain of histopathology specimens should be performed as well as culture of tissue. Other sites of concomitant non-CNS infection may include lungs, bone marrow, liver, and spleen, which could also be evaluated to establish a tissue diagnosis. Less common sites include intra-abdominal or peripheral lymph nodes, skin, mucous membranes, gastrointestinal tract, adrenal glands, bones, joints, and the genitourinary tract.

## 3. Treatment and Subsequent Diagnostic Testing

With the development of effective antifungal therapy, histoplasmosis infection of the central nervous system has been transformed from a universally fatal illness to a manageable condition, if identified early. The cornerstone of treatment for severe infection with histoplasmosis continues to consist of amphotericin B (AmB) [23]. The liposomal preparation of AmB (Ambisome^®^) is preferred, because of the decreased nephrotoxicity compared with the traditional deoxycholate formulation [24] and improved clinical response with statistically significant improvement in survival when studied for induction therapy in patients with AIDS excluding those with CNS involvement [25]. Furthermore, liposomal AmB is known to penetrate CNS structures well. In an experimental rabbit model of *Candida albicans* CNS infection, liposomal AmB at a dose of 5 mg/kg/day was found to generate peak levels capable of sterilizing brain tissue. In contrast, the colloidal dispersion (ABCD or Amphotec^®^) and lipid complex formulations of AmB (Abelcet^®^) did not generate effective levels and did not sterilize brain tissue [26]. The clinical efficacy of lipid complex formulations of AmB for treatment of CNS histoplasmosis in humans has also been shown to be poor. In the largest most recently published study case series, one-year survival of patients treated with liposomal AmB was 80.8%, compared with 56.2% for those treated with lipid complex AmB. When the mean survival time was compared, those patients treated with liposomal AmB or deoxycholate AmB survived 10.6 ± 0.5 months compared with 8.3 ± 1.1 months if treated with lipid complex AmB (*p* = 0.040) [11].

**Figure 1:**
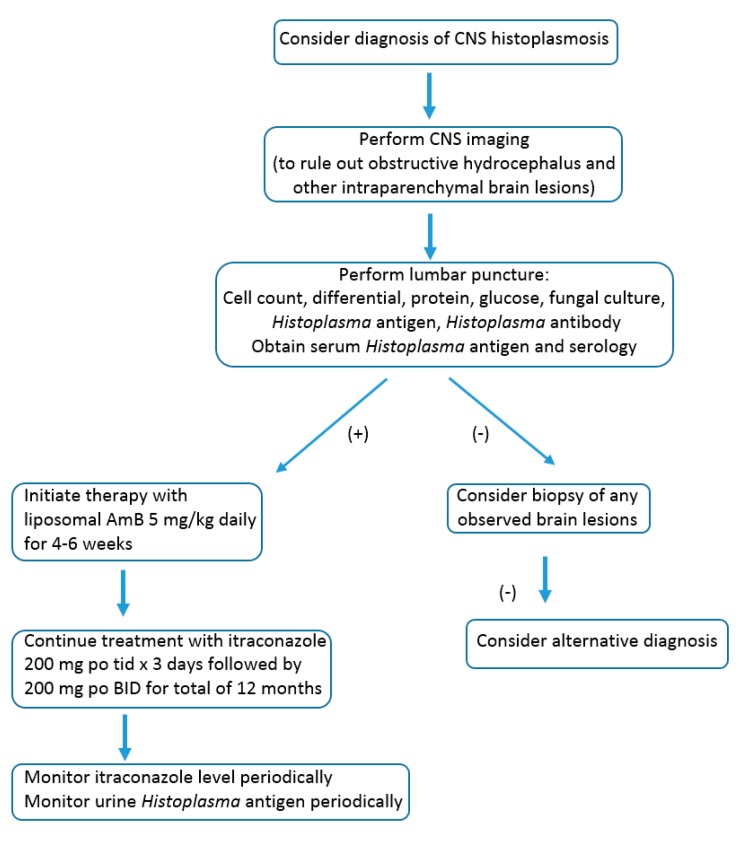
Testing and treatment algorithm.

After initial treatment with liposomal AmB, 5 mg/kg daily for a duration of up to 4–6 weeks as tolerated, a transition to itraconazole is recommended for at least 12 months (Figure 1) [23]. Furthermore, because of the risk of relapse, it is recommended to re-evaluate CSF parameters prior to discontinuation of itraconazole at 12 months to ensure that the cell counts have normalized and the *Histoplasma* antigen in the CSF is negative. During the course of treatment with itraconazole, it is important to monitor drug levels to ensure that appropriate absorption is occurring. Random serumlevels >1 and <5 mcg/mL measured by high pressure liquid chromatography (HPLC) using the combination of itraconazole and hydroxyl itraconazole (an active metabolite) have been suggested by some authors and should be initially measured after 14 days of treatment [23,27]. Some experts have suggested targeting higher trough levels of 2 mcg/mL for more serious invasive infections (LJW, personal communication). The upper value of the serum level of itraconazole, which is more likely to produce toxicity, was found to be around 17 mcg/mL by bioassay measurement or approximately 4 mcg/mL by HPLC, the assay most often used) [28]. Using a loading dose of oral liquid itraconazole (200 mg three times per day for three days), followed by 200 mg twice per day, will help reach goal levels more quickly. Formulations of itraconazole that are currently available to treat CNS infection include itraconazole tablets (Sporanox^®^ or generic version) and liquid itraconazole. Tablets require an acid stomach environment for optimal absorption, whereas the liquid formulation is more reliably absorbed [27]. In dogs and cats with fungal infection, the generic form of the itraconazole tablet appears to achieve equivalent levels [29]. However, clinically, there have been 3 cases of failure of the generic form of itraconazole with lower drug levels measured [30]. The newly approved SUBA-itraconazole formation, although with improved absorption compared to itraconazole tablets, has not been studied in histoplasmosis meningeal infection and therefore is not recommended [31,32].

Responses to treatment with other triazoles as initial or follow up therapy for CNS infection with histoplasmosis have been variable. Some authors have reported clinical success, with initial therapy consisting of fluconazole or itraconazole [33,34], however, others have reported failures [35], In an animal model of CNS histoplasmosis, fluconazole was inferior to deoxycholate AmB, even though the levels of fluconazole measured in the CNS were higher [36], AmB or itraconazole was not detectable at significant levels in the brain tissue of mice in this study; however, hydroxy-itraconazole was detectable at low levels. Interestingly, brain fungal burden was similar when itraconazole monotherapy was compared with AmB in this study. The fact that MICs of *Histoplasma* to fluconazole are higher may explain why, despite higher CNS concentration, fluconazole failures are observed [37]. In addition, resistance to fluconazole has been demonstrated to have developed in relapse isolates from 10 out of 17 patients with AIDS and disseminated histoplasmosis [38].

The activity of newer triazoles has also been studied in small case series and in vitro. Based on the available data, it appears that voriconazole is less effective than posaconazole, based on increased MICs and resistance to voriconazole in patients with AIDS, wherein fluconazole failed in the treatment of histoplasmosis [37]. When isavuconazole was later studied in this same group of isolates, no significant increase in MIC was observed to this agent [39]. *Histoplasma* MICs to isavuconazole were also quite low, at ≤0.007. In addition, limited studies have shown that isavuconazole penetrates well into brain tissue [40,41], whereas posaconazole penetration is low [42,43]. However, posaconazole has been clinically reported to successfully manage histoplasmosis in cases that have failed with other treatments. In one small case series, 6 patients responded favorably (5 patients with disseminated disease, including one with CNS infection) to treatment with posaconazole after a failure to respond to other agents [44]. In a study examining the use of isavuconazole for initial therapy in patients with endemic fungal infections, 4 out of 7 patients with histoplasmosis had a successful outcome at the end of treatment [45]. In this group, there was one patient with CNS infection who had a favorable response. Therefore, in cases of failure or intolerance to itraconazole during treatment for CNS histoplasmosis, it remains unclear which of the newer alternative triazoles (isavuconazole or posaconazole) is best, based on the very limited clinical experience available.

Experts recommend that treatment should be continued for at least one year, at which time CSF cell count, chemistry, and antigen should be measured [23]. Discontinuation of treatment can be considered if the antigen in CSF is negative and other clinical and imaging abnormalities have resolved or are greatly improved and stabilized. Lifelong treatment may be required in immunocompromised patients in whom immune function cannot be improved, such as patients with HIV infection and AIDS, with persistent low CD4 count, or patients with solid organ transplantation or bone marrow transplantation requiring significant levels of immunosuppression.

Patients who have had CNS histoplasmosis should be followed for at least 3 years for recurrence. Lumbar puncture should be performed to assess cell count, protein and glucose, and antigen concentration if recurrent clinical symptoms develop or CNS imaging abnormalities worsen. Monitoring antigen clearance from CSF is an important marker for assessing the response to treatment (unpublished observation, L.J. Wheat).

## 4. Summary

With the advent of effective antifungal therapy, central nervous system infection with histoplasmosis has been transformed from a universally fatal complication to a condition that can be effectively managed in most cases. For clinicians, maintaining a high index of suspicion is critical, particularly when evaluating cases of subacute meningitis in which a specific infectious etiology has not been identified or cases of disseminated histoplasmosis with central nervous system symptoms. It is important to note that both immune compromised and non-immune compromised individuals are at risk. A diagnosis can be best achieved by testing urine, blood, and CSF for the presence of the *Histoplasma* antigen. In resource limited settings, ELISA assays have been developed that are highly sensitive and specific, but have not been extensively evaluated using CSF. Preferred initial therapy consists of liposomal AmB 5 mg/kg for 4–6 weeks, followed by treatment with a loading dose of oral itraconazole 200 mg three times daily for three days, and followed by 200 mg twice daily to achieve levels of >1 and <5 mcg/mL for at least 12 months. The liquid formulation of itraconazole is preferred because of improved absorption compared to capsules, however, some patients may find the liquid formulation difficult to tolerate. In resource limited settings, the amphotericin B deoxycholate formulation may need to be substituted for liposomal AmB. Although there is minimal clinical data available, isavuconazole or posaconazole are likely the best alternatives in cases of intolerance to itraconazole when treating CNS histoplasmosis. The *Histoplasma* serum or urinary antigen (if previously positive), as well as itraconazole drug levels, should be periodically followed after the initiation of treatment. At the end of 12 months of therapy, it should be confirmed that the CSF is cleared of *Histoplasma* antigens, with normal cell counts, prior to discontinuation of therapy. Outcomes in the most recent case series were very good, with most patients surviving [11]. Relapses occur in cases of poor adherence to treatment, particularly in settings of immune compromise, such as AIDS.

## Figures and Tables

**Table 1:** Summary of most common clinical characteristics of patients with CNS histoplasmosis (modified from reference [11]).

Clinical Characteristic	Percent of Cases
Immune compromised	71%
Typical symptoms of meningitis	86%
Symptoms for more than 2 weeks	65%
Lymphocytic CSF pleocytosis	50%
Mass lesion on CNS imaging	20%

**Table 2:** Diagnostic tests in CSF for *Histoplasma* meningitis.

Test	Sensitivity	Specificity
ID or CF antibody [17]	22/43 (51%)	22/23 (96%)
Cytopathology-CSF [11]	2/11 (18%)	Not done
Histopathology-Brain [11]	6/11 (55%)	Not done
Culture-CSF [17]	9/47 (19%)	119/119 (100%)
Culture-Brain [11]	2/4 (50%)	Not done
*Histoplasma* antigen EIA * [17]	36/50 (78%)	140/154 (97%)
IgG or IgM anti-*Histoplasma* antibody EIA * [17]	37/45 (82%)	142/153 (93%)
antigen or anti-*Histoplasma* antibody EIA * [17]	48/49 (98%)	139/153 (91%)

* MiraVista Diagnostics, Indianapolis, IN, USA.
